# Comparison of Efficacy and Safety of Baloxavir and Oseltamivir in Children With Influenza: A Systematic Review and Meta-Analysis

**DOI:** 10.7759/cureus.71289

**Published:** 2024-10-12

**Authors:** Jay Manuel, Kaushikkumar S Barot, Abshiro H Mayow, Dhruvi Modi, Muhammad Tariq, Jawad Hussain, Muhammad Daniyal Waheed, Samina Kutiyana

**Affiliations:** 1 Medicine, Louisiana State University Health Sciences Center, New Orleans, USA; 2 Pediatrics, Shantabaa Medical College and General Hospital, Amreli, IND; 3 Medicine, St. George's University School of Medicine, St. George's, GRD; 4 Internal Medicine, Gujarat Adani Institute of Medical Sciences, Bhuj, IND; 5 Pediatrics, Children Hospital Pakistan Institute of Medical Sciences, Islamabad, PAK; 6 Medicine, Sahara Medical College, Narowal, PAK; 7 Internal Medicine, Foundation University Medical College, Islamabad, PAK; 8 Cardiology, Mahatma Gandhi Memorial Hospital, Bhojpur, NPL

**Keywords:** baloxavir, children, influenza, oseltamivir, review article

## Abstract

This systematic review and meta-analysis compared the efficacy and safety of baloxavir marboxil and oseltamivir in treating influenza in children. A comprehensive literature search was conducted across multiple databases, identifying five studies (four observational and one randomized controlled trial) with a pooled sample size of 2,261 patients. The analysis revealed that baloxavir marboxil significantly reduced the duration of fever compared to oseltamivir (mean difference: -13.49 hours, 95% CI: -23.75 to -3.24). However, there was no significant difference in the time to resolution of overall influenza symptoms between the two treatments (mean difference: -4.55 hours, 95% CI: -19.48 to 10.37). Safety analysis, though limited by available data, suggested a lower incidence of nausea and vomiting with baloxavir marboxil compared to oseltamivir. Both drugs demonstrated comparable safety profiles for other adverse events. These findings indicate that while both medications remain viable options for managing pediatric influenza, baloxavir marboxil may offer advantages in terms of rapid fever reduction and potentially fewer gastrointestinal side effects. However, the study highlights the need for more robust, large-scale randomized controlled trials focusing exclusively on pediatric populations to strengthen the evidence base. Clinicians should consider individual patient factors, local resistance patterns, and current guidelines when making treatment decisions. Future research should explore combination therapies and their potential to manage severe influenza cases in children and conduct more comprehensive safety assessments in pediatric populations.

## Introduction and background

Influenza, commonly referred to as the flu, is a highly contagious viral respiratory illness that poses a significant health burden, particularly in children [1]. The disease is caused by influenza viruses, which are categorized into types A, B, C, and D, with types A and B being primarily responsible for seasonal epidemics [2]. In children, influenza can result in a range of symptoms, from mild respiratory distress to severe complications such as pneumonia, bronchitis, sinus infections, and even encephalopathy [3,4]. Due to the underdeveloped immune systems in younger children, they are more vulnerable to severe forms of the disease, leading to increased rates of hospitalization and, in rare cases, death [5]. Given the unpredictability of influenza virus strains, antiviral treatment has become an essential component in managing the disease, particularly in reducing the duration of illness and mitigating the risk of complications [6]. However, the efficacy and safety of antiviral agents can vary, necessitating a thorough comparison of available treatments in children. Two antiviral agents, baloxavir marboxil and oseltamivir, are widely used to treat influenza [7]. However, their comparative efficacy and safety in children remain a subject of debate. 

Oseltamivir, a neuraminidase inhibitor, has been a mainstay of influenza treatment for nearly two decades. It works by preventing the release of new viral particles from infected cells, thus limiting the spread of the virus within the host [8,9]. Baloxavir marboxil, a cap-dependent endonuclease inhibitor, is a novel antiviral drug with a unique mechanism of action. It inhibits viral replication at an earlier stage of the viral life cycle by blocking the initiation of mRNA synthesis, thereby reducing viral load more rapidly than neuraminidase inhibitors like oseltamivir [10,11]. 

While both drugs are effective in treating influenza, there are critical differences in their safety profiles. Oseltamivir is generally well tolerated, but common side effects include nausea, vomiting, and neuropsychiatric events, which are of particular concern in children. Baloxavir, though promising in efficacy, may also have unique adverse effects such as diarrhea and elevated liver enzymes [12]. Furthermore, the emergence of baloxavir-resistant strains raises questions about its long-term efficacy, particularly in pediatric populations [13]. Given the importance of public health in reducing influenza-related morbidity in children, this systematic review and meta-analysis aims to provide a comprehensive comparison of the efficacy and safety of baloxavir marboxil and oseltamivir. By synthesizing current evidence, we hope to guide clinicians in making informed treatment decisions for pediatric influenza management. 

## Review

Methodology 

Search Strategy 

A comprehensive literature search was conducted to identify relevant studies comparing the efficacy and safety of baloxavir marboxil and oseltamivir in children with influenza. Databases including PubMed, Embase, Cochrane Central Register of Controlled Trials (CENTRAL), and ClinicalTrials.gov were systematically searched from inception to September 2024. Search terms used included a combination of medical subject headings (MeSH) and keywords, such as “baloxavir,” “oseltamivir,” “influenza,” “children,” “pediatrics,” and “antiviral therapy.” No language restrictions were applied. Additionally, reference lists of all relevant articles and previous reviews were manually searched to ensure the inclusion of any additional eligible studies. The search was conducted independently by two authors. Any disagreements between the two authors were resolved through discussion, and the principal author was involved if required. This meta-analysis was performed and reported as per the guidelines of Preferred Reporting of Systematic Review and Meta-analysis (PRISMA). 

Study Selection 

The selection process was carried out in two stages. First, titles and abstracts were independently screened by two reviewers to identify studies meeting the predefined inclusion criteria. Eligible studies included randomized controlled trials (RCTs) or observational studies comparing baloxavir marboxil and oseltamivir in children aged ≤18 years with confirmed influenza. Studies were excluded if they focused solely on adult populations, did not provide separate data for pediatric patients, or involved other antiviral agents. Full texts of the selected studies were then reviewed in detail to confirm eligibility. Any disagreements during the selection process were resolved through discussion or consultation with a principal investigator. 

Data Extraction and Outcomes 

Data extraction was performed independently by two reviewers using a standardized data collection form. The following information was extracted from each study: author details, year of publication, study design, sample size, patient demographics, and treatment regimens. Key outcomes included time to resolution of fever, time to resolution of influenza symptoms (such as cough, fatigue, and nasal congestion), and safety events (including adverse drug reactions, gastrointestinal disturbances, and neuropsychiatric events). In cases of missing or unclear data, corresponding authors were contacted for clarification. Any disagreements during the data extraction process were resolved through discussion or consultation with a principal investigator. 

Data Analysis 

The data were synthesized using RevMan version 5.4 (The Cochrane Collaboration, London, England, GBR). Continuous outcomes, such as time to resolution of fever and time to resolution of influenza symptoms, were pooled and expressed as mean differences (MD) with 95% confidence intervals (CI). For dichotomous safety outcomes, such as the occurrence of adverse events, risk ratios (RR) with 95% CIs were calculated. Heterogeneity between studies was assessed using the I² statistic, with a value greater than 50% indicating substantial heterogeneity. A random-effects model was applied where significant heterogeneity was present; otherwise, a fixed-effects model was used. This methodological approach ensured a rigorous and transparent assessment of the comparative efficacy and safety of baloxavir marboxil and oseltamivir in children with influenza. 

Results 

**Figure 1:**
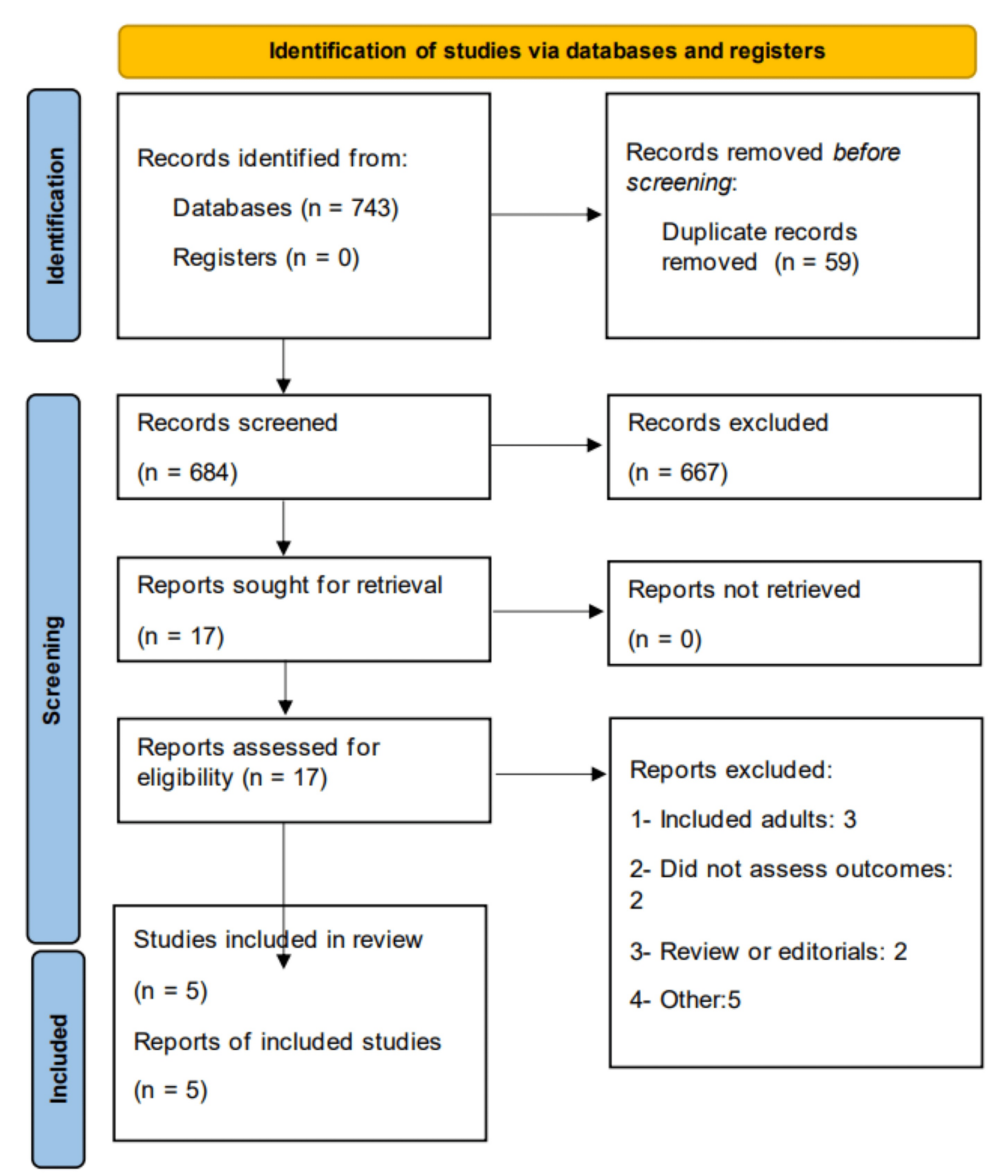
PRISMA flowchart of study selection PRISMA: Preferred Reporting of Systematic Review and Meta-analysis

Figure 1 shows the PRISMA flowchart of study selection. A total of 743 records were identified through the initial search. After removing duplicates and irrelevant studies, 684 studies underwent title and abstract screening. Of these, the full texts of 17 studies were reviewed for eligibility. Ultimately, five studies met the inclusion criteria, comprising four observational studies and one RCT. Table 1 shows the characteristics of the included studies. The RCT was multinational, while two of the observational studies were conducted in China and two in Japan. The pooled sample size across the included studies was 2,261 patients, with 1,154 patients receiving baloxavir marboxil and 1,107 patients receiving oseltamivir. Table 2 and Table 3 present the quality assessment of the included observational studies and the RCT, respectively.

**Table 1:** Study characteristics RCT: Randomized controlled trial

Author	Study design	Region	Groups	Sample size	Results summary
Baker et al., 2023 [14]	RCT	Multinational	Baloxavir marboxi	79	The study included 94 children aged five to 11 years (61 baloxavir, 33 oseltamivir) with similar baseline characteristics. Both treatments showed comparable safety profiles and efficacy in alleviating symptoms, with baloxavir demonstrating a shorter median time to cessation of fever.
			Oseltamivir	39	
Ge et al., 2024 [15]	Observational	China	Baloxavir marboxi	420	Baloxavir demonstrated a comparable duration for fever reduction to oseltamivir. The results indicate that baloxavir is well-tolerated and effective in children aged five to 11 years, with a notably shorter time to cessation of viral shedding compared to oseltamivir.
			Oseltamivir	445	
Nezu et al., 2023 [16]	Observational	China	Baloxavir marboxi	555	Baloxavir significantly reduced fever duration compared to oseltamivir in early childhood influenza, with 99.6% of baloxavir-treated patients experiencing fever resolution within one day vs. 1.1% for oseltamivir.
			Oseltamivir	556	
Saito et al., 2020 [17]	Observational	Japan	Baloxavir marboxi	34	The duration of fever and symptoms in baloxavir-treated children with influenza A/H1N1pdm09 and A/H3N2 did not significantly differ from those treated with oseltamivir.
			Oseltamivir	17	
Wagatsuma et al., 2022 [18]	Observational	Japan	Baloxavir marboxi	66	The study found that baloxavir-treated children had a marginally shorter fever duration compared to oseltamivir-treated children However, the duration of overall symptoms did not significantly differ between the baloxavir and oseltamivir groups.
			Oseltamivir	50	

**Table 2:** Quality assessment of observational studies The quality assessment was done using the Newcastle-Ottawa scale.

Author ID	Selection of participant	Comparability between groups	Outcome and exposure assessment	Overall quality score	Quality grade
Ge et al., 2024 [15]	4	2	3	9	Good
Nezu et al., 2023 [16]	4	2	3	9	Good
Saito et al., 2020 [17]	3	2	3	8	Good
Wagatsuma et al., 2022 [18]	3	1	3	7	Good

**Table 3:** Quality assessment of RCT This quality assessment was done using the Cochrane risk of bias assessment tool. RCT: Randomized controlled trial

Author ID	Randomization	Concealment	Blinding of participants and personnel	Blinding of outcome assessor	Incomplete outcome data	Selective reporting	Other bias
Baker et al., 2023 [14]	No	No	No	No	Unclear	Unclear	No bias

Comparison of the Duration of Fever Between the Two Groups (in Hours)

**Figure 2:**
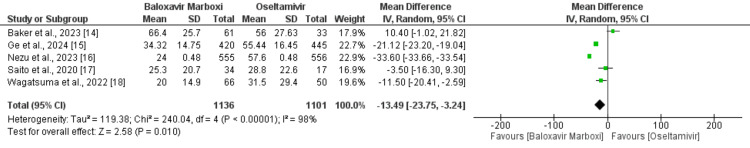
Comparison of the mean duration of fever between the two groups References [14-18]

We included five studies comparing the mean duration of fever between the baloxavir marboxil and oseltamivir groups. The results of the pooled analysis are shown in Figure 2. The mean duration of fever after receiving the drug was significantly lower in patients receiving baloxavir marboxil compared to oseltamivir (MD: -13.49, 95% CI: -23.75 to -3.24). High heterogeneity was reported among the study results (I^2^: 98%).

Comparison of the Amount of Time for the Resolution of Influenza Symptoms Between the Two Groups (in Hours)

**Figure 3:**
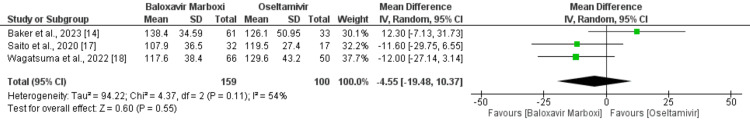
Comparison of time taken for the resolution of symptoms between the two groups References [14,17,18]

Three studies were included in the pooled analysis of comparing the time of resolution of influenza symptoms between baloxavir marboxil and oseltamivir groups, and the results are depicted in Figure 3. The mean time of resolution of influenza symptoms was not significantly different between the two groups (MD: -4.55, 95% CI: -19.48 to 10.37). Significant heterogeneity was reported among the study results (I^2^: 54%). 

Safety Analysis 

Only two out of the five studies provided data on adverse events for both baloxavir marboxil and oseltamivir groups, limiting our ability to perform a pooled analysis. Both studies demonstrated a significantly higher incidence of nausea and vomiting in patients treated with oseltamivir compared to those receiving baloxavir marboxil. Specifically, in the study by Ge et al., 12.13% of patients on oseltamivir experienced nausea and vomiting, compared to 2.38% of those on baloxavir (p < 0.001). Baker et al. also reported a higher rate of vomiting in the oseltamivir group (18%) compared to the baloxavir group (5%). However, no significant differences were observed between the two groups for other adverse events, including dizziness, headache, diarrhea, and rash. Overall, both studies indicated a comparable safety profile for most adverse events, except for the higher occurrence of nausea and vomiting in the oseltamivir group. 

Discussion 

This systematic review and meta-analysis provide valuable insights into the comparative efficacy and safety of baloxavir marboxil and oseltamivir in pediatric influenza treatment. Our findings suggest that baloxavir marboxil may offer a significant advantage in reducing fever duration in children with influenza. However, the lack of substantial difference in overall symptom resolution time between the two treatments indicates that both drugs remain viable options for managing influenza in pediatric populations. We also found that in terms of safety, baloxavir marboxil has a lower risk of nausea and vomiting, showing a better safety profile of the drug compared to oseltamivir. 

Interestingly, earlier studies focusing on pediatric influenza cases showed different results. These results are consistent with both the overall trial population and previous baloxavir studies [19-21]. A randomized controlled trial involving children aged one to 12 years found no significant differences in fever duration (41.2 hours for baloxavir vs. 46.8 hours for oseltamivir) or symptom duration (66.4 vs. 67.9 hours) for influenza A infections [19]. Similarly, an observational study in Japan during the 2018-2019 influenza season reported comparable fever durations between baloxavir and oseltamivir treatments in children with influenza A [22]. However, our findings are more consistent with a study conducted on Chinese adolescents and adults with influenza A. This research demonstrated a significantly shorter fever duration in the baloxavir group (1.5 days, range 1.0-2.5) compared to the oseltamivir group (2.5 days, range 1.5-3.0), with a p-value below 0.001 [13]. Regarding viral load reduction, baloxavir exhibited a more rapid decrease in infectious viral titer compared to oseltamivir. This was evidenced by 'time to cessation of viral shedding' (TCVS) measurements (approximately one day for baloxavir vs. three days for oseltamivir) and changes from baseline in influenza titer. 

Our review of safety outcomes, particularly gastrointestinal adverse events, aligns with findings from previous research. A notable study by Liu et al. [23] compared various antiviral treatments for influenza. Their analysis revealed that oseltamivir, when administered at 75 mg twice daily, was associated with a higher incidence of nausea (based on moderate-quality evidence) and vomiting (based on high-quality evidence) compared to placebo. Interestingly, Liu et al. also found that both zanamivir and baloxavir were linked to significantly fewer instances of nausea when compared to oseltamivir. Additionally, peramivir, at a 300 mg dosage, showed a lower frequency of vomiting [23]. While our study didn't conduct a pooled analysis of safety events due to the limited number of available studies, our narrative review uncovered similar trends to those reported by Liu et al. [23]. However, it's important to note a key difference in study populations. Liu et al.'s [23] research included RCTs involving both adult and pediatric patients, whereas our analysis focused exclusively on studies conducted on children. This distinction in study populations underscores the need for more comprehensive, pediatric-specific research to fully understand the safety profiles of these antiviral medications in children. Our findings, while consistent with broader population studies, highlight the importance of age-specific considerations in influenza treatment strategies. 

The present meta-analysis has certain limitations. First, only five studies were included, of which only one was an RCT, with the rest being observational studies associated with potential selection bias. More RCTs conducted exclusively on children are needed to strengthen the evidence base. Second, we were unable to perform a pooled analysis of safety outcomes due to the limited number of studies reporting comprehensive safety data. These limitations underscore the need for further research to confirm our findings and establish more robust safety profiles for both treatments in pediatric populations. 

The findings of this meta-analysis have significant research and clinical implications. From a research perspective, our study highlights the need for more robust, large-scale RCTs focusing exclusively on pediatric populations. Such studies would help clarify the comparative efficacy and safety profiles of baloxavir marboxil and oseltamivir in children. Clinically, our results suggest that baloxavir marboxil may be preferable for rapid fever reduction in pediatric influenza cases. However, the similar overall symptom resolution times indicate that both drugs remain valuable treatment options. The potentially lower incidence of gastrointestinal side effects with baloxavir marboxil may be particularly beneficial for children. Nonetheless, clinicians should consider individual patient factors, local resistance patterns, and updated guidelines when making treatment decisions. Future research should also explore combination therapies and their potential in managing severe influenza cases in children. 

## Conclusions

This meta-analysis comparing baloxavir marboxil and oseltamivir in pediatric influenza treatment reveals important insights. Baloxavir marboxil demonstrates a significant advantage in reducing fever duration, although overall symptom resolution time is similar between the two drugs. Safety profiles are comparable, with baloxavir potentially offering a lower risk of gastrointestinal side effects, particularly nausea and vomiting. However, the limited number of studies, especially RCTs, highlights the need for more robust research in pediatric populations. While both drugs remain viable treatment options, baloxavir's rapid fever reduction and potentially better tolerability may make it preferable in certain cases. Clinicians should consider individual patient factors, local resistance patterns, and current guidelines when making treatment decisions. Future research should focus on large-scale pediatric trials and explore combination therapies for severe influenza cases in children.
